# Supplementary material for: Empirical methods for controlling false positives and estimating confidence in ChIP-Seq peaks
Source: BMC Bioinformatics. 2008 Dec 5;9:523. doi: 10.1186/1471-2105-9-523 (PMC2628906; doi:10.1186/1471-2105-9-523)
Supplement: Additional File 1 — A variety of html documents from the USeq web site detailing the available applications, their best usage, output file type descriptions, command line menus, etc. [file 1471-2105-9-523-S1.zip › USeqUserGuides/JavaDoc.zip!/JavaDoc/trans/bkgrnd/LoadedOligo.html]

LoadedOligo


|  |  |  |
| --- | --- | --- |
| **Overview** \| **Package** \| **Class** \| **Use** \| **Tree** \| **Deprecated** \| **Index** \| **Help** | |  |
| **PREV CLASS**   **NEXT CLASS** | **FRAMES**    **NO FRAMES**     **All Classes** |  |
| SUMMARY: NESTED \| FIELD \| CONSTR \| METHOD | DETAIL: FIELD \| CONSTR \| METHOD |  |


---


## trans.bkgrnd Class LoadedOligo

```
java.lang.Object
  trans.bkgrnd.LoadedOligo
```

**All Implemented Interfaces:**: java.lang.Comparable

---

``` public class LoadedOligo extends java.lang.Object implements java.lang.Comparable ```

BPMap line contain gc and tm calculations.

---

**Constructor Summary**

`LoadedOligo(java.lang.String line)`

`LoadedOligo(java.lang.String chromosome,
int startPosition,
java.lang.String sequence,
double intensityPM,
double intensityMM,
double tm,
double gc)`


| **Method Summary** | |
| --- | --- |
| `int` | `compareTo(java.lang.Object obj)` |
| `java.lang.String` | `getChromosome()` |
| `double` | `getGc()` |
| `double` | `getIntensityMM()` |
| `double` | `getIntensityPM()` |
| `int` | `getNumberMisMatches()` |
| `java.lang.String` | `getSequence()` |
| `int` | `getStartPosition()` |
| `double` | `getTm()` |
| `void` | `setIntensityPM(double intensityPM)` |

**Methods inherited from class java.lang.Object**

`equals, getClass, hashCode, notify, notifyAll, toString, wait, wait, wait`

**Constructor Detail**

### LoadedOligo

```
public LoadedOligo(java.lang.String chromosome,
                   int startPosition,
                   java.lang.String sequence,
                   double intensityPM,
                   double intensityMM,
                   double tm,
                   double gc)
```

---


### LoadedOligo

```
public LoadedOligo(java.lang.String line)
```


**Method Detail**

### compareTo

```
public int compareTo(java.lang.Object obj)
```

:   **Specified by:**: `compareTo` in interface `java.lang.Comparable`

---


### getChromosome

```
public java.lang.String getChromosome()
```

---


### getGc

```
public double getGc()
```

---


### getIntensityMM

```
public double getIntensityMM()
```

---


### getIntensityPM

```
public double getIntensityPM()
```

---


### getSequence

```
public java.lang.String getSequence()
```

---


### getStartPosition

```
public int getStartPosition()
```

---


### getTm

```
public double getTm()
```

---


### getNumberMisMatches

```
public int getNumberMisMatches()
```

---


### setIntensityPM

```
public void setIntensityPM(double intensityPM)
```


---


|  |  |  |
| --- | --- | --- |
| **Overview** \| **Package** \| **Class** \| **Use** \| **Tree** \| **Deprecated** \| **Index** \| **Help** | |  |
| **PREV CLASS**   **NEXT CLASS** | **FRAMES**    **NO FRAMES**     **All Classes** |  |
| SUMMARY: NESTED \| FIELD \| CONSTR \| METHOD | DETAIL: FIELD \| CONSTR \| METHOD |  |


---
